# Influence of Immobilization, Stretching, and Activity on Isometric Muscle Strength and Gait in Young People with Spastic Cerebral Palsy

**DOI:** 10.3390/jcm15103869

**Published:** 2026-05-18

**Authors:** Martin Svehlik, Andreas Habersack, Bernhard Guggenberger, Nina Mosser, Markus Tilp, Tanja Kraus, Annika Kruse

**Affiliations:** 1 Department of Orthopaedics and Trauma, Medical University of Graz, Auenbruggerplatz 34, 8036 Graz, Austria; martin.svehlik@medunigraz.at (M.S.); andreas.habersack@uni-graz.at (A.H.); bernhard.guggenberger2@fh-joanneum.at (B.G.); tanja.kraus@medunigraz.at (T.K.); 2 Department of Human Movement Science, Sport and Health, University of Graz, Aigner-Rollett-Allee 39, 8010 Graz, Austria; nina.mosser@uni-graz.at (N.M.); markus.tilp@uni-graz.at (M.T.); 3 Institute of Physiotherapy, JOANNEUM University of Applied Sciences, Alte Poststraße 149, 8020 Graz, Austria

**Keywords:** cerebral palsy, orthotic treatment, equinus, gait, strength

## Abstract

**Background/Objectives**: Neurological impairments in children with Cerebral Palsy (CP) often lead to altered muscle architecture and function, resulting in calf muscle contractures. Orthotic immobilization aims to promote muscle–tendon unit lengthening through sustained stretch but may also induce disuse atrophy. This study investigated whether combining immobilization with daily activity yields different effects on muscle strength and gait function compared with immobilization alone. **Methods:** Fourteen ambulant children with spastic CP and equinus deformity (8 unilateral, 6 bilateral; mean age 9.93 ± 3.0 years; GMFCS I: 10, GMFCS II: 4) participated in a 12-week randomized controlled trial. Participants were assigned to either continuous immobilization (23 h/day) using a dynamic ankle–foot orthosis or a combined protocol consisting of 14 h/day immobilization and 10 h/day of activity involving ankle mobility and calf muscle activation. Outcomes included isometric muscle strength, joint range of motion, gait parameters, and functional measures (Gait Outcomes Assessment List (GOAL) and the Paediatric Outcome Data Collection Instrument (PODCI)). Data were analyzed using linear mixed models with Bonferroni correction. **Results**: Significant time effects were observed for the knee angle at initial contact (IC), the ankle angle at IC, maximum dorsiflexion, and maximum dorsiflexion during swing. A significant group × time interaction was found only for hindfoot-tibia angle at IC. Within-group improvements were noted in activities of daily living, body image and self-esteem, and basic mobility. No significant changes were found for muscle strength or for most questionnaire subscales. **Conclusions**: The findings indicate time-related improvements in gait, with no consistent advantage of the combined intervention. Further studies with larger samples are needed to evaluate potential long-term effects.

## 1. Introduction

Cerebral palsy (CP) is the most common neuromuscular disorder in children. Spastic CP is the predominant subtype and is defined by elevated muscle tone, impaired selective motor control, and structural alterations in muscle–tendon units [1,2]. The abnormal muscle activity imposes excessive mechanical load on the developing skeleton, promoting secondary bony deformities and joint instability. The interplay of these factors leads to substantial impairments in mobility, particularly in walking function, making CP the major cause of physical disability in childhood. At the muscular level, children with CP demonstrate greater relative fascicle excursions for the same absolute length change during everyday movements compared to typically developing children [3], markedly diminishing their active force capacity [4,5,6]. These functional alterations are associated with typical gait deviations, such as equinus at initial contact, reduced dorsiflexion during swing, decreased stride length, slower walking speed, and diminished push-off power [7]. Deviations in gait kinematics are frequently accompanied by kinetic abnormalities. Reduced strength of the plantarflexors leads to lower ankle moments and diminished power output during terminal stance, thereby increasing the metabolic cost of walking and limiting overall function [8,9]. Compensatory strategies at the knee and hip, such as increased hip flexion and knee hyperextension, are commonly present and may further aggravate musculoskeletal problems over time [10].

One of the main therapeutic strategies for children with CP involves managing calf muscle contractures, which are a major cause of equinus gait. Recent studies have further advanced the understanding of contracture development in children with cerebral palsy, emphasizing that contractures are not solely the result of neural hyperactivity but also of altered muscle growth and musculoskeletal development [11,12,13,14]. Impaired longitudinal muscle growth, reduced sarcomere addition, and increased extracellular matrix deposition have been identified as key contributors to progressive muscle–tendon shortening [12,15]. In addition, reduced voluntary muscle activation and chronic positioning in shortened muscle lengths may further exacerbate these structural adaptations during growth [13,16,17]. Together, these mechanisms highlight the complex interaction among neural impairment, mechanical loading, and growth-related processes in the development of equinus deformity [11,17]. Spasticity of the calf muscles and equinus gait are commonly addressed through immobilization, typically achieved with orthotic devices. Ankle-foot orthoses (AFOs) are widely employed to enhance and maintain ankle range of motion during functional tasks such as walking [18]. Numerous gait analysis studies have demonstrated that AFOs improve gait speed, cadence, stride length, ankle dorsiflexion at initial contact and during swing, and knee motion [19,20,21].

In this context, the duration of immobilization represents a crucial aspect of orthotic treatment in individuals with CP. Children often wear orthoses both during the day and at night to manage spastic equinus. However, extended immobilization is known to provoke disuse atrophy, which is reflected in reductions in muscle mass, thickness, strength, and myofiber area, alongside increased muscle protein degradation [11,22,23]. Such atrophy may be particularly detrimental in children exhibiting equinus gait, as this gait pattern partly compensates for plantarflexor weakness by utilizing elastic energy storage [24]. Prolonged immobilization may not only alter muscle properties but also remove this compensatory mechanism and heighten eccentric loading [25]. In children with CP, weakness of the plantarflexors interacts with gait pathology by reducing push-off power and increasing dependency on passive elastic components [24]. The optimal orthotic regimen should therefore be sufficiently long to induce functional improvements, e.g., increased ankle dorsiflexion, but short enough to allow active muscle contractions and avoid muscle atrophy. Nevertheless, despite the widespread use of orthotic interventions, there is limited evidence regarding the most effective duration, orthosis type, and integration of functional load. A better understanding of these factors is essential for enhancing gait efficiency, preventing secondary musculoskeletal deformities, and informing clinical decision-making.

This study aimed to gain information about the effects of orthotic treatment on the spastic plantarflexor muscles in children and adolescents with CP. Two orthotic protocols were compared. The conventional protocol consisted of a dynamic AFO worn during both daytime and nighttime to provide continuous stretching through immobilization (Immobilization Group—IG). The alternative protocol combined immobilization with active plantarflexor engagement by means of an innovative orthotic design (Immobilization/Activity Group—IAG). We hypothesized that both orthotic protocols would lead to improvements in functional outcomes, including improved ankle dorsiflexion at initial contact and during swing phase. Furthermore, we hypothesized that the IAG would not show a decline in isometric muscle strength compared to the IG.

## 2. Materials and Methods

### 2.1. Study Design

This study is part of a larger research project investigating the impact of immobilization, stretching, and physical activity on the characteristics of spastic muscles in children with cerebral palsy. The sample size calculation for the overall project was based on gastrocnemius medialis fascicle length, as previously described [2], and resulted in a total sample size of 14 subjects, i.e., 7 per group. The trial was prospectively registered at ClinicalTrials.gov (NCT05269745). The study protocol was approved by the Ethics Committee of the Medical University of Graz (approval number 32-115 ex 19/20), and written consent was obtained in advance from the parents or guardians of all participating children and adolescents. This randomized controlled trial was designed and reported in accordance with the Consolidated Standards of Reporting Trials (CONSORT) guidelines. A completed CONSORT checklist is provided as Supplementary Material.

### 2.2. Participants

The participants (Table 1) were recruited through the orthopaedic department of the affiliated university hospital. All included children were ambulatory, capable of understanding and following verbal instructions, and presented with restricted ankle range of motion (RoM), defined clinically as maximal dorsiflexion ≤5° with extended knees, which was assessed in advance by a physician. Every participant was able to ambulate for at least short distances without walking aids and was classified within GMFCS levels I to III. None of the 14 participants had undergone surgery in their lifetime, and none had received a Botulinum toxin A injection within 12 months prior to the measurements. Children were excluded if they had any type of CP other than spastic type or were receiving oral antispastic or muscle relaxant medication. Selective voluntary motor control of the lower limbs was evaluated using the Selective Control Assessment of the Lower Extremity (SCALE) [1]. The assessment of SCALE was administered by a trained clinician with experience in paediatric gait analysis and motor function assessment in children with cerebral palsy. All assessments were performed according to the standardized SCALE protocol. Randomized allocation into the IG or the IAG was stratified according to Gross Motor Function Classification System level (I + II vs. III) and age category (5–10 years vs. 11–15 years).

**Figure 1:**
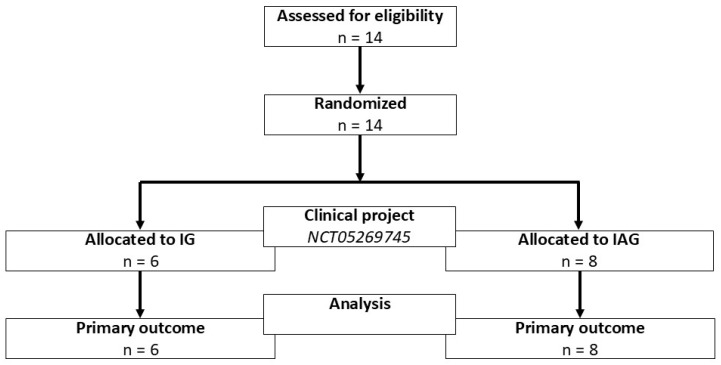
Overview of participant flow. IG: Immobilization Group; IAG: Immobilization/Activity Group.

Participants were randomly assigned to one of the two intervention groups in a 1:1 allocation ratio. Randomization was performed using a computer-generated stratified block procedure by the principal investigator and the investigator who performed the assessments. Group assignments were concealed in sealed, opaque envelopes, with no access for other study personnel. Finally, due to limited personnel resources, blinding to group allocation was not feasible in this study. An overview of participant flow, including enrolment, allocation, and follow-up, is provided in Figure 1.

### 2.3. Intervention Protocol

**Figure 2:**
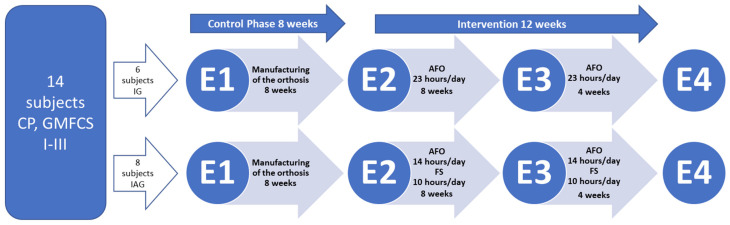
Study timeline and assessment schedule. Fourteen ambulatory children and adolescents with spastic cerebral palsy (CP) and equinus deformity were randomly assigned to the Immobilization Group (IG) or the Immobilization/Activity Group (IAG). The main difference between groups was the duration of active time wearing only the foot shell (FS). The control phase lasted 8 weeks, followed by a 12-week intervention. Assessments were conducted at four time points: E1—before device fabrication; E2—pre-intervention; E3—after 8 weeks of intervention; E4—after 12 weeks of intervention. Abbreviations: FS—foot shell; AFO—ankle-foot orthosis; CP—cerebral palsy; GMFCS—Gross Motor Function Classification System [2].

Participants in the IG received a conventional treatment protocol, consisting of a dynamic AFO worn continuously during both day and night (23 h daily). Children assigned to the IAG were provided with the same model of AFO at night for approximately 8 h and for an additional 6 h during daytime, amounting to a total orthosis wear time of 14 h per day. For the remaining 10 h of the day, individuals in the IAG wore only the orthotic foot shell (FS), without the proximal calf section, to maintain proper foot positioning while allowing unrestricted ankle movement and ongoing correction of foot deformities such as pes equinovarus, pes equinovalgus, or midfoot collapse. The term “activity” in the IAG refers to habitual daily movement during FS wear, including spontaneous walking and routine functional activities. No structured or supervised exercise program was prescribed during this period. Prior to initiating the orthotic intervention, an eight-week control period was implemented, during which customized orthoses were fabricated for each participant. This was followed by a 12-week intervention phase during which the prescribed orthotic protocols were applied. Therefore, the total intervention duration was 12 weeks and was divided into an initial 8-week phase (E2–E3) and a subsequent 4-week phase (E3–E4), as illustrated in Figure 2. A 12-week intervention period was selected based on previous studies demonstrating that skeletal muscle structural adaptations to altered mechanical loading occur within several weeks [26]. With regard to orthotic treatment, significant changes in GM fascicle length were reported after approximately 16 ± 4 weeks [22]. Therefore, a 12-week duration was considered sufficient to capture clinically relevant adaptations in muscle architecture while remaining feasible in a pediatric clinical setting. Muscle functional performance, and mechanical parameters were evaluated at four separate time points: prior to orthotic fabrication, at the onset of orthotic use, and then after 8 weeks and again after 12 weeks of orthotic treatment. An 8-week control phase preceded the intervention period. This phase was implemented to allow for orthosis fabrication, individual fitting, and stabilization of baseline conditions prior to the start of treatment. During this period, no intervention-related orthotic training effects were expected. The 8-week control phase (E1–E2) was therefore considered a preparatory period for orthosis fabrication and baseline stabilization and was not included as a separate intervention condition in the statistical analysis. An overview of the study design is depicted in Figure 2.

To ensure adherence to the prescribed orthotic wearing schedule (Figure 2), each orthosis was equipped with a monitoring sensor (Orthotimer, Balingen, Germany). The sensor was embedded in the soft lining of the orthosis and recorded the skin temperature at 15-min intervals. These data were retrieved after completion of the intervention using a wireless reader and analyzed digitally. This approach made it possible to objectively determine wearing compliance. Additionally, a daily activity log was provided in which parents documented the times during which the FS was worn and recorded the type of activities undertaken during wear.

**Figure 3:**
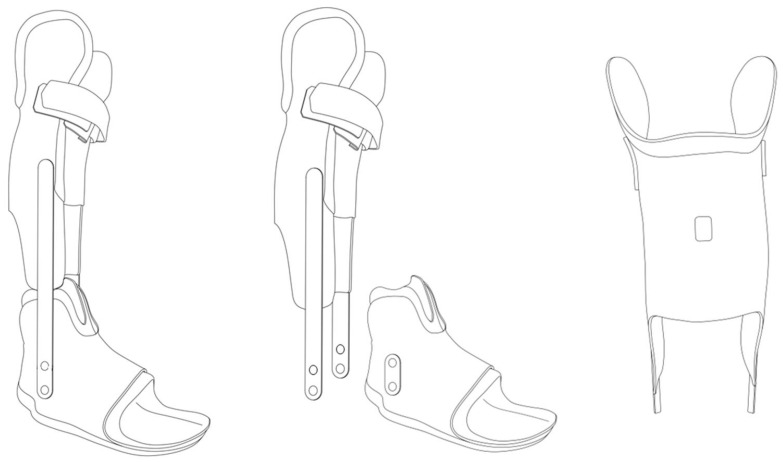
Schematic example of an orthosis for the Immobilization/Activity Group (IAG) (**middle**) and Immobilizaton Group (IG) (**left**). The orthoses were equipped with an Orthotimer sensor (**right**) (Orthotimer, Balingen, Germany). The IAG used the same type of orthosis as the IG; however, the lower leg shell was removable to allow for calf muscle activity (middle) [2].

Each participant allocated to one of the two groups received a custom-made AFO (Figure 3). The orthosis was designed not only to correct the ankle equinus position but also to address deformities involving the Chopart and subtalar joints. The inner layer was made of a soft material to ensure user comfort, whereas the outer component consisted of a rigid laminate structure composed of matrix layers and multiple fiber composites, forming both the calf and foot sections. The lower-leg portion featured an s-shaped calf shell with condylar support, and the FS offered circular stabilization of the foot. Subtalar motion was restricted by a circumferential frame, and the heel was secured with a detachable heel cap. Both components (FS and lower-leg section) were connected via a fixed metal ankle hinge positioned at maximum passive dorsiflexion with the knee fully extended but without causing discomfort.

The innovative aspect of the orthosis in the IAG was the modular connection between the foot and calf components, which allowed the FS to be used independently of the lower-leg section. This configuration enabled immobilization and stretching of the plantarflexors to treat equinus and correct foot deformities, while still allowing unrestricted ankle motion during the active part of the intervention. As a result of this design, participants in the IAG were able to engage the plantarflexors more actively during daytime periods.

### 2.4. Data Collection and Processing

#### 2.4.1. Ankle Range of Motion and Isometric Muscle Strength

To evaluate the muscle strength of the participants, isometric maximum voluntary contractions (IMVCs) of the plantarflexor muscles were assessed using an isokinetic dynamometer (CON-TREX MJ, CMV AG, Duebendorf, Switzerland) [3]. The foot of the more affected leg of the participant was securely positioned on the footplate, ensuring alignment between the centre of the ankle joint and the axis of rotation of the dynamometer. Two submaximal trials were performed beforehand to provide familiarization with the procedure, supported by real-time visual feedback of the generated torque. Following this, IMVCs lasting 5 s were conducted at an ankle joint angle corresponding to 50% of each child’s individual RoM. Isometric strength was therefore assessed at 50% of the individual range of motion. This testing position was chosen to ensure a standardized and functionally relevant mid-range joint position across participants with varying degrees of joint mobility. Mid-range testing is widely used in pediatric and clinical biomechanics to balance mechanical advantage, reduce end-range variability, and improve between-subject comparability in longitudinal assessments [27,28]. The ankle joint RoM was determined in advance using the dynamometer [3,4]. A rest period of 2 min was given between trials to minimize the influence of fatigue. For further analysis, the peak isometric torque was used. Peak torque values were normalized to body mass.

#### 2.4.2. 3D Gait Analysis

Gait analysis was performed using a 10-camera motion capture system (Qualisys, Gothenburg, Sweden) together with five force plates embedded in the walkway (AMTI, Watertown, MA, USA). Marker placement followed the calibrated anatomical systems technique (CAST) protocol. In addition, the Oxford Foot Model (OFM) [5] was applied to obtain detailed foot and ankle kinematics. Kinematic parameters of the pelvis, hip, knee, and ankle joints, as well as kinetic joint moments and powers, were calculated using Qualisys Track Manager and Visual3D (C-Motion Inc., Germantown, MD, USA). Moments and power values were normalized to body weight. All participants walked barefoot at a self-selected comfortable speed along a 10-m walkway. Only trials with regular, uninterrupted walking were included. For each participant, at least three valid trials per side with a clean force plate contact were recorded. Three-dimensional kinematic and kinetic parameters of the hip, knee, and ankle, together with spatiotemporal data (e.g., gait velocity, cadence, and stride length), served as outcome measures.

#### 2.4.3. Participation Questionnaires

The Gait Outcomes Assessment List (GOAL) [6] was used as an assessment questionnaire for ambulant children and adolescents with CP. The GOAL is a valid instrument to explore a child’s gait function and performance using 48 items. The GOAL questionnaire ensures an assessment of all domains of the International Classification of Functioning, Disability and Health: body structure, function, and participation [6]. In addition, the Paediatric Outcome Data Collection Instrument (PODCI) [7] was also used. The PODCI is a standard instrument to quantify functional health (e.g., mobility) and treatment efficacy in patients aged 2–18 years with a wide range of diagnoses. The PODCI scale has demonstrated reliability, sensitivity, and validity of the assessment of the functional health of the patients [7,8]. The parents or caregivers of the participants completed both questionnaires at each measurement time point (Figure 2).

### 2.5. Statistical Analyses

All statistical analyses were conducted using SPSS (version 26, SPSS Inc., Chicago, IL, USA). The significance level was set to *p* = 0.05. Data were tested for normality using the Shapiro–Wilk test, histograms, and Q-Q plots. A linear mixed model with comparison of the main effects and Bonferroni correction was performed to assess the effects of the orthotic treatment. The within-subjects factor was time, with four levels: familiarisation before the manufacturing, FAM), before the intervention (PRE), after 8 weeks of the intervention (POST), and after 12 weeks of the intervention (FOLLOW). “FAM” is defined as the familiarization measurement conducted prior to the control phase, during which participants became familiar with all measurement procedures (including gait analysis and strength testing) to ensure standardized conditions and reduce potential learning effects in subsequent assessments. These time points were explicitly incorporated into the statistical analysis as the within-subject factor “time” in the linear mixed model, allowing for the evaluation of longitudinal changes across the entire study period, including the control and intervention phases. The between-subjects factor was orthosis group, with two levels: IG and IAG. The measurement obtained at the end of the control phase (E2; pre-intervention time point) served as the baseline reference for all subsequent analyses. The longitudinal changes across the intervention period were assessed using a repeated-measures linear mixed model including all scheduled time points. Orthosis wearing time is reported as a descriptive adherence measure for the full study cohort and was not included as a group-comparative outcome in the statistical analysis. Accordingly, no inferential statistical testing (F-values or *p*-values) was performed for orthosis wearing time.

## 3. Results

Significant effects were found in the measured wearing time between PRE-POST and POST-FOLLOW (*p* = 0.01, Cohen’s d = 0.77), and between the measured time and the self-assessed daily activity diary for PRE-POST (*p* = 0.014, Cohen’s d = 0.73) and POST-FOLLOW (*p* = 0.014, Cohen’s d = 0.73) (Table 2).

No significant differences were observed between the FAM and PRE time points for any outcome (all *p* > 0.05), confirming baseline stability prior to the intervention.

No statistically significant main effects for time or group × time interaction were found for maximum dorsiflexion (MDF), maximum plantarflexion (MPF), or RoM (Table 3). The linear mixed model indicated F-values between 0.3 and 1.7 with *p* > 0.1 for all comparisons. Normalized isometric strength also showed no significant changes over time or between groups.

However, significant within-group improvements were observed for Activities of Daily Living (F(3,28) = 2.92, *p* = 0.05) and Body Image & Self-Esteem (F(3,28) = 4.16, *p* = 0.01) determined with the (GOAL), and the Transfer Basic Mobility Scale—Standardized Mean (F(3,28) = 3.73, *p* = 0.02) determined with the PODCI (Table 4). No significant changes were found for the remaining GOAL or PODCI subscales.

Three participants from the IG and two from the IAG were excluded from the analysis of the kinematic gait parameters. For kinetic parameters, one additional participant from the IG and two from the IAG were excluded due to incorrect data recording relating to subject compliance. To assess potential selection bias, baseline characteristics of included and excluded participants were compared, revealing no statistically significant differences between groups.

For the gait parameters (Table 5), significant effects of time were found for the knee angle at initial contact (IC_Knee) (F(3,21.00) = 3.08, *p* = 0.05), for the angle between the hindfoot and the tibia at initial contact (IC_HindfootTibia) (F(3,18.98) = 3.34, *p* = 0.04), maximum dorsiflexion of the hindfoot relative to the tibia (MaxDorsi_HindfootTibia) (F(3,19.34) = 3.12, *p* = 0.05), and maximum dorsiflexion during swing of the hindfoot relative to the tibia (MaxDorsi_swing_HindfootTibia) (F(3,19.17) = 3.59, *p* = 0.03). A significant group × time interaction effect was observed for IC_HindfootTibia (F(3,18.98) = 3.87, *p* = 0.03). No other statistically significant main or interaction effects were found for the ankle, knee, or forefoot–hindfoot kinematics as well as the kinetics (all *p* > 0.05).

## 4. Discussion

This study investigated the biomechanical and functional effects of a short-term orthotic intervention in children with spastic CP. The primary finding was that no significant differences between the two orthotic protocols were detected for nearly all outcome parameters, despite the marked difference in daily wearing time. As the comparison of both protocols represented the main aim of the study, this finding is particularly relevant. It suggests that shorter daily orthosis use may be sufficient to achieve clinically meaningful improvements. The present findings provide clinically relevant insights into the effects of different “doses” of orthotic treatment. While continuous immobilization aims to maximize passive stretching, the combination of reduced immobilization with active muscle use may offer a more balanced approach by promoting both structural adaptation and preservation of muscle function. From a clinical perspective, this may support the consideration of orthotic concepts that integrate periods of active movement rather than relying solely on prolonged immobilization. This is of clinical importance, as the optimal duration of daily orthotic treatment is still unclear and remains debated in routine practice. A more moderate wearing schedule may therefore improve treatment acceptance, adherence, and long-term feasibility [9]. Given the exploratory nature of this analysis and the absence of a specific sample size calculation for gait-related outcomes, all findings should be interpreted as hypothesis-generating. The observed changes provide descriptive insight into potential treatment effects of the orthotic interventions but are not intended to serve as confirmatory evidence.

While no significant changes were observed for isolated ankle kinematics or overall ankle range of motion, several time-dependent changes were detected in other gait parameters. Across both groups, a significant reduction in knee flexion at initial contact was observed over the intervention period, indicating a more extended knee posture during early stance. Such adaptations may reflect improved limb positioning at foot strike and a more stable loading response, which are relevant features in children with equinus gait and may contribute to a reduction in excessive crouch-like mechanics during initial stance.

In addition, significant time effects were found for hindfoot-tibia kinematics, including hindfoot–tibia alignment at initial contact, maximum dorsiflexion during stance, and maximum dorsiflexion during swing. These findings suggest that the intervention was associated with subtle changes in distal foot–ankle alignment during key phases of the gait cycle. Increased dorsiflexion relative to the tibia during stance and swing may reflect improved functional positioning of the hindfoot, potentially facilitating toe clearance and a more favourable foot placement at ground contact. Such adaptations have previously been reported following orthotic management of equinus gait, where improvements in overall locomotor patterns may occur even in the absence of measurable changes in isolated ankle joint RoM [10]. However, the observed improvements in gait parameters should be interpreted cautiously, as their magnitude may not translate into noticeable functional benefits in daily life.

Importantly, the hindfoot-tibia angle at initial contact also demonstrated a significant group × time interaction. The group means indicate that the IG showed a progressive shift towards a more dorsiflexed hindfoot position at initial contact over time, whereas this pattern was not consistently observed in the IAG. This suggests that continuous immobilization may have produced a slightly greater change in hindfoot positioning at foot strike, possibly reflecting more sustained passive stretching of the plantarflexor–Achilles tendon complex. However, given the small sample size and variability within the groups, this interaction should be interpreted cautiously. Overall, the results indicate that the orthotic intervention primarily affected global gait alignment and distal foot positioning rather than isolated ankle joint motion. Forefoot–hindfoot kinematic variables derived from the Oxford Foot Model were not further discussed, as they did not demonstrate consistent or interpretable intervention-related changes in Chopart- or subtalar joint-related kinematic.

Ankle power during terminal stance showed no significant changes over time. This absence of change may be related to the unchanged isometric plantarflexor strength observed in the present study, suggesting that functional push-off capacity was not substantially altered within the 12-week intervention period. Given the close coupling between plantarflexor strength and ankle power generation, the lack of measurable changes in strength may help explain the stable power outcomes. However, we note that inter-individual variability and the limited sample size for gait analysis may have also reduced the sensitivity to detect subtle changes in push-off mechanics.

In addition to the observed kinematic changes, significant improvements were noted in self-reported daily activities and self-esteem, as well as in transfers and basic mobility in both groups. These results suggest that orthotic interventions can provide meaningful functional and psychosocial benefits, even when objective biomechanical changes are modest. This is consistent with previous reports indicating that functional outcomes and patient-perceived improvements often respond more sensitively to orthotic treatment than isolated kinematic parameters [6,18]. Interestingly, improvements in PODCI basic mobility scores were observed despite minimal changes in objective gait parameters, suggesting that perceived functional gains may precede measurable biomechanical adaptations. Increased independence in activities of daily living and enhanced self-image are highly relevant clinical outcomes, particularly for children and adolescents, who frequently experience limitations in participation and self-efficacy. However, neither of the two orthotic approaches appears to be more beneficial in this context. While statistically significant improvements were observed in selected subscales, these findings should be interpreted within the broader context of the biomechanical results. In particular, the observed improvements in gait-related parameters over time may be associated with enhanced functional performance in daily activities, as reflected in the GOAL and PODCI outcomes. The integration of objective and subjective measures nevertheless suggests that changes at the biomechanical level may translate, at least in part, into perceived functional benefits. However, these relationships should be interpreted with caution, as the present study was not designed to establish relationships and/or causal links between biomechanical changes and patient-reported outcomes. Future studies with larger cohorts are needed to further investigate relationships and to better understand the interaction between mechanical adaptations and patient-reported outcomes.

A notable finding of this study is the objectively measured wearing time obtained via the Orthotimer, which revealed a substantial decline in orthosis use over the intervention period and towards follow-up. These objective data suggest that the actual treatment exposure was lower than intended, particularly at later stages, which may help explain the limited ankle-level biomechanical effects observed. In contrast, parent-reported wearing times consistently overestimated orthosis use, in line with recent compliance research [9]. This discrepancy underscores the importance of objective adherence monitoring, as reliance on subjective reports alone may lead to an overestimation of treatment intensity and a potential misinterpretation of intervention effects.

Contrary to our initial hypothesis, normalised isometric plantarflexor strength did not change significantly over time in the IG and did not differ between groups after the intervention. This may be explained by several factors. First, the 12-week intervention duration may have been insufficient to induce measurable atrophic changes. In addition, children with cerebral palsy may rely on compensatory neuromuscular strategies that preserve force output despite reduced activity. Another factor is that the use of isometric testing at a single joint angle may not have been sensitive enough to detect subtle changes in muscle function. However, this stability is a positive finding, given that prolonged immobilization or constraint-based interventions have previously been associated with muscle atrophy or reductions in force-generating capacity in both animal [19,20] and human studies [21]. In contrast, the protocols used did not induce any measurable decrements, suggesting that moderate wearing durations may minimise the risks associated with disuse while still providing therapeutic benefits. However, the observed changes in normalized isometric plantarflexor strength should be interpreted cautiously, as the study was not specifically powered to detect between-group differences in strength outcomes. The absence of a detectable strength decline in the IG should be interpreted in the context of several further potential influencing factors. Although disuse atrophy is a well-established theoretical concern during prolonged immobilization, the present findings may reflect a more complex interaction of mechanisms. The intervention duration, while sufficient to induce changes in muscle architecture, may not have been long enough to produce measurable alterations in isometric strength, particularly in a paediatric population, where ongoing growth and neuromuscular maturation may influence adaptation processes. Furthermore, compensatory neuromuscular mechanisms and lower compliance with orthosis therapy may contribute to the preservation of strength despite reduced mechanical loading, as has been suggested in children with cerebral palsy. In addition, the sensitivity of isometric strength measurements to detect subtle longitudinal changes may be limited by variability in participant performance and challenges related to voluntary activation in this population. Taken together, these considerations suggest that the present findings should not be interpreted as contradicting the concept of disuse-related adaptations, but rather as highlighting the multifactorial nature of strength responses under clinical and developmental conditions. Improvements in the stiffness or extensibility of the plantarflexor muscles, as described in previous studies [3,22,23], may have contributed indirectly to improved initial contact posture by enabling the dorsiflexor muscles to act more effectively, even in the absence of measurable RoM changes.

The findings of this study should be interpreted in light of several methodological limitations. In particular, the relatively small sample size and the heterogeneity inherent in children with CP may limit the generalizability of the results and reduce the statistical power to detect subtle group differences. The present investigation was embedded within this larger study framework and therefore used the same available cohort. Due to the strict inclusion criteria, the long-term intervention protocol, and the complexity of repeated gait laboratory measurements, recruitment was limited. All eligible and available participants within the study period were therefore included. Although the sample size was calculated based on gastrocnemius fascicle length as part of a broader project, it may not be sufficient to detect changes in the primary outcomes of the present study (isometric strength and gait parameters). A separate a priori sample size calculation specifically for gait-related outcomes was not performed, as these analyses were secondary and exploratory within the overarching study design. Therefore, the study should be considered exploratory with respect to between-group comparisons. Furthermore, although the randomized controlled design strengthens internal validity, the observed variability in adherence and individual responses may have influenced the magnitude of the effects. Therefore, the present findings should be interpreted with caution, and no definitive conclusions regarding the superiority of one intervention over the other can be drawn. Although the randomized allocation and repeated measurements strengthen the internal validity of the study, the variability typically observed in children with CP, including differences in motor impairment, gait patterns, and selective motor control, may have masked potential group-specific effects. Although the use of objective orthosis-wearing sensors provided valuable insight into treatment adherence, the observed discrepancies between measured and reported wearing times highlight the challenges of accurately monitoring orthotic compliance in pediatric populations. These factors should be taken into account when interpreting the results and when designing future studies on orthotic management in children with CP. A limitation of this study is the presence of baseline differences between the intervention groups, with participants in the IAG being, on average, older, taller, heavier, and presenting more frequently with bilateral involvement compared to the IG. Although randomization was applied, such imbalances can occur in studies with relatively small sample sizes. These differences may have influenced between-group comparisons and should be considered when interpreting the results. A further limitation is the reduced sample size for the gait analysis, as 5 out of 14 participants had to be excluded due to incomplete or unusable gait data (e.g., technical issues or inability to complete the measurement protocol). Given the proportion of participants excluded from both the kinematic (36%) and kinetic (57%) gait analyses, these results should be interpreted with caution and considered exploratory. This resulted in a small final sample, which limits the generalizability of the findings and precludes confirmatory statistical conclusions. However, the longitudinal within-subject design with repeated assessments across multiple time points still allows for a descriptive evaluation of individual responses and temporal trends. Therefore, the results of the gait analysis should be interpreted as exploratory and hypothesis-generating. Gait velocity, cadence, and stride length were recorded as part of the gait analysis protocol but were not included in the inferential statistical analysis due to high inter-trial variability and reduced data completeness following quality control procedures. Therefore, the main gait outcomes were restricted to kinematic and kinetic variables with sufficient data robustness across participants. Moreover, the 12-week intervention period was chosen based on previous evidence indicating that structural and functional adaptations in skeletal muscle can occur within this timeframe under altered mechanical loading conditions in children with cerebral palsy [22]. While this timeframe is sufficient to capture early treatment-related changes, longer-term follow-up would be valuable to assess the persistence, progression, or potential reversal of strength adaptations beyond the intervention period.

In summary, our findings suggest that orthotic treatment strategies for equinus gait may not require near-continuous daily wearing to achieve functional improvements. A more moderate, individualised wearing schedule may reduce the burden and might improve the adherence without compromising the treatment outcomes. The observed functional gains further support the notion that orthoses may exert benefits beyond isolated joint mechanics, influencing proximal gait behaviour and daily functional participation. Future work should explore whether combining orthotic treatment with physiotherapy, strength training or task-specific gait retraining could amplify these effects [11,29]. Furthermore, future research should focus on larger, adequately powered studies to confirm the exploratory findings of the present investigation. In addition, longer-term follow-up is warranted to evaluate the durability and potential delayed effects of orthotic treatment on muscle function and gait parameters. The integration of objective adherence monitoring tools may further improve the accuracy of treatment evaluation by reducing reliance on self-reported data. Moreover, future methodological developments should aim to enhance the efficiency and reliability of muscle–tendon unit assessment, including the potential implementation of automated or semi-automated tracking techniques. Collectively, these directions may help refine orthotic treatment strategies and improve their clinical applicability.

## 5. Conclusions

Moderate-duration orthotic intervention in children with spastic CP and equinus gait was associated with meaningful functional and psychosocial improvements, even in the absence of measurable changes in ankle range of motion or plantarflexor strength. Importantly, no significant differences between the two orthotic protocols were observed for the majority of outcome parameters, indicating that the Immobilization/Activity approach was not superior to immobilization alone with regard to the assessed biomechanical and functional outcomes. Subtle kinematic adaptations, particularly in hindfoot–tibia alignment at initial contact, suggest improvements in distal foot positioning that may contribute to more efficient gait mechanics. Notably, comparable outcomes were achieved despite the reduced daily wearing time in the Immobilization/Activity approach. This finding suggests that incorporating periods of active ankle use may allow a reduction in overall orthosis wearing time without compromising treatment effects, potentially improving treatment acceptance and long-term adherence while limiting risks associated with prolonged immobilization.

## Figures and Tables

**Table 1:** Participant characteristics of the children and adolescents with spastic cerebral palsy in the Immobilization Group (IG) and the Immobilization/Activity Group (IAG). Data is presented as mean (SD).

Participants’ Characteristics	IG	IAG
Number	6	8
Gender (female/male)	2/4	2/6
Age (years)	9.0 (2.7)	10.6 (2.9)
USCP/BSCP	3/3	5/3
Body mass (kg)	25.7 (5.0)	45.8 (17.0)
Body height (cm)	130.7 (12.5)	147.5 (16.3)
Lower leg length (cm)	30.0 (3.0)	34.3 (3.4)
**Clinical Characteristics**		
GMFCS Level I/II	3/3	6/2
SCALE (points: 2/1/0)	0/6/0	1/7/0

Grading of the Selective Control Assessment of the Lower Extremity (SCALE) is presented for the more affected leg or in children with bilateral spastic cerebral palsy (BSCP) and for the affected leg in those with unilateral spastic cerebral palsy (USCP), respectively. Grading was limited to the ankle joint: normal: 2 points; impaired: 1 point; unable: 0 points.

**Table 2:** Wearing time of the orthoses, measured by a sensor (Orthotimer, Balingen, Germany) and by a daily activity log documented by the parents. Data are presented as the mean between the respective two time points (SD).

	Orthotimer			Daily Activity Log			Diff Orthotimer—Activity Log	
	PRE-POST	POST-FOLLOW	Δ Diff	PRE-POST	POST-FOLLOW	Δ Diff	PRE-POST	POST-FOLLOW
Lower Leg Shell [h]	7.83 (4.81)	5.73 (6.29)	−2.1	9.92 (4.88)	8.76 (7.23)	−1.16	−21.07%	−34.59%
Foot Shell [h]	8.31 (3.08)	4.92 (4.44)	−3.39	15.09 (3.47)	11.93 (6.86)	−3.16	−44.94%	−58.76%

**Table 3:** Ankle joint function and isometric muscle strength in children with spastic cerebral palsy. Negative ankle values correspond to a plantarflexed position. Isometric strength is additionally normalized to body mass. Data are presented as the mean (SD).

Parameter	Effect	Group	FAM	95% CI	PRE	95% CI	POST	95% CI	FOLLOW	95% CI	DoF	F	Sig.
MDF [°]	Time	IG	4.58 (4.75)	[−5.11, 14.27]	5.44 (4.75)	[−4.25, 15.13]	7.76 (4.75)	[−1.93, 17.45]	7.06 (4.75)	[−2.63, 16.75]	30.90	1.39	0.26
	Time × Group	IAG	−2.44 (3.76)	[−10.11, 5.23]	−3.96 (3.76)	[−11.63, 3.71]	1.13 (3.86)	[−6.74, 9.00]	0.54 (3.86)	[−7.33, 8.41]	30.90	0.19	0.91
MPF [°]	Time	IG	−48.80 (2.71)	[−54.33, −43.27]	−51.22 (2.71)	[−56.75, −45.69]	−52.26 (2.71)	[−57.79, −46.73]	−50.44 (2.71)	[−55.97, −44.91]	31.13	0.32	0.81
	Time × Group	IAG	−53.74 (2.14)	[−58.10, −49.38]	−52.30 (2.14)	[−56.66, −47.94]	−52.56 (2.22)	[−57.09, −48.03]	−51.56 (2.22)	[−56.09, −47.03]	31.13	0.92	0.44
RoM [°]	Time	IG	53.38 (5.55)	[42.06, 64.70]	56.66 (5.55)	[45.34, 67.98]	60.02 (5.55)	[48.70, 71.34]	57.50 (5.55)	[46.18, 68.82]	30.70	1.04	0.39
	Time × Group	IAG	51.30 (4.39)	[42.34, 60.26]	48.34 (4.39)	[39.38, 57.30]	53.63 (4.53)	[44.39, 62.87]	52.04 (4.53)	[42.80, 61.28]	30.70	0.41	0.75
Norm. isometric strength [Nm/kg]	Time	IG	0.73 (0.18)	[0.35, 1.11]	0.70 (0.16)	[0.36, 1.04]	0.66 (0.15)	[0.34, 0.98]	0.63 (0.15)	[0.31, 0.95]	16.39	0.96	0.43
	Time × Group	IAG	0.96 (0.34)	[0.25, 1.67]	0.61 (0.13)	[0.34, 0.88]	0.47 (0.13)	[0.20, 0.74]	0.61 (0.13)	[0.34, 0.88]	18.25	0.69	0.51

MDF: maximum dorsiflexion; MPF: maximum plantarflexion; RoM: range of motion; Norm: normalized; IG: Immobilization Group; IAG: Immobilization/Activity Group; CI: confidence interval; DoF: degrees of freedom.

**Table 4:** Results of the Gait Outcomes Assessment List (GOAL) and the Paediatric Outcome Data Collection Instrument (PODCI). Data are presented as the mean (SD). Bold numbers: significant difference (*p* < 0.05).

Parameter	Effect	Group	FAM	95% CI	PRE	95% CI	POST	95% CI	FOLLOW	95% CI	DoF	F	Sig.
GOAL													
A_Activities_of_Daily_Living	Time	IG	82.72 (5.71)	[71.08, 94.36]	81.98 (5.71)	[70.34, 93.62]	87.65 (5.71)	[76.01, 99.29]	88.64 (5.71)	[77.00, 100.28]	31.09	2.92	0.05
	Time × Group	IAG	90.74 (4.52)	[81.52, 99.96]	86.88 (4.52)	[77.66, 96.10]	95.67 (4.65)	[86.19, 105.15]	93.56 (4.65)	[84.08, 103.04]	31.09	0.20	0.90
B_Gait_Function_Mobility	Time	IG	75.20 (6.60)	[61.74, 88.66]	79.80 (6.60)	[66.34, 93.26]	78.20 (6.60)	[64.74, 91.66]	77.40 (6.60)	[63.94, 90.86]	30.96	0.72	0.55
	Time × Group	IAG	81.00 (5.22)	[70.35, 91.65]	82.63 (5.22)	[71.98, 93.28]	87.28 (5.41)	[76.25, 98.31]	88.13 (5.41)	[77.10, 99.16]	30.96	0.46	0.72
C_Pain_Discomfort_Fatigue	Time	IG	88.57 (6.94)	[74.40, 102.74]	91.90 (6.94)	[77.73, 106.07]	93.06 (6.94)	[78.89, 107.23]	89.39 (6.94)	[75.22, 103.56]	30.23	0.13	0.94
	Time × Group	IAG	88.52 (5.48)	[77.33, 99.71]	82.77 (5.75)	[71.03, 94.51]	86.95 (5.76)	[75.19, 98.71]	90.16 (5.76)	[78.40, 101.92]	30.23	0.49	0.69
D_Physical_Activities_Sports_Recreation	Time	IG	50.08 (11.71)	[26.19, 73.97]	59.19 (11.71)	[35.30, 83.08]	53.81 (11.71)	[29.92, 77.70]	58.33 (11.71)	[34.44, 82.22]	30.91	0.96	0.42
	Time × Group	IAG	62.76 (9.26)	[43.87, 81.65]	64.27 (9.26)	[45.38, 83.16]	72.27 (9.45)	[52.99, 91.55]	56.14 (9.45)	[36.86, 75.42]	30.91	1.79	0.17
E_Gait_Pattern_Appearance	Time	IG	66.11 (7.31)	[51.21, 81.01]	67.78 (7.31)	[52.88, 82.68]	61.11 (7.31)	[46.21, 76.01]	67.22 (7.31)	[52.32, 82.12]	31.31	0.04	0.99
	Time × Group	IAG	61.81 (5.78)	[50.03, 73.59]	60.14 (5.78)	[48.36, 71.92]	68.77 (6.09)	[56.35, 81.19]	63.61 (6.09)	[51.19, 76.03]	31.31	0.81	0.50
F_Use_of_Braces_Mobility_Aids	Time	IG	45.00 (9.46)	[25.57, 64.43]	40.21 (11.58)	[16.43, 63.99]	50.00 (9.46)	[30.57, 69.43]	65.00 (9.46)	[45.57, 84.43]	26.48	0.58	0.63
	Time × Group	IAG	50.00 (7.48)	[34.64, 65.36]	60.27 (9.92)	[39.90, 80.64]	56.73 (7.89)	[40.53, 72.93]	47.21 (7.89)	[31.01, 63.41]	26.48	2.09	0.13
G_Body_Image_Self_Esteem	Time	IG	50.00 (8.54)	[32.59, 67.41]	51.67 (8.54)	[34.26, 69.08]	64.17 (8.54)	[46.76, 81.58]	67.50 (8.54)	[50.09, 84.91]	31.42	4.16	**0.01**
	Time × Group	IAG	45.83 (6.75)	[32.07, 59.59]	47.40 (6.75)	[33.64, 61.16]	58.66 (7.06)	[44.27, 73.05]	58.66 (7.06)	[44.27, 73.05]	31.42	0.08	0.97
Standardized_TOTAL_Score	Time	IG	71.06 (6.37)	[58.07, 84.05]	71.11 (6.37)	[58.12, 84.10]	73.58 (6.37)	[60.59, 86.57]	75.27 (6.37)	[62.28, 88.26]	30.99	1.45	0.25
	Time × Group	IAG	73.40 (5.04)	[63.12, 83.68]	72.77 (5.04)	[62.49, 83.05]	80.17 (5.16)	[69.65, 90.69]	76.37 (5.16)	[65.85, 86.89]	30.99	0.36	0.78
Upper_Extremity_Scale_Standardized_Mean	Time	IG	85.00 (5.09)	[74.62, 95.38]	85.83 (5.09)	[75.45, 96.21]	87.38 (5.09)	[77.00, 97.76]	95.00 (5.09)	[84.62, 105.38]	31.09	1.96	0.14
	Time × Group	IAG	89.06 (4.67)	[79.54, 98.58]	90.92 (4.67)	[81.40, 100.44]	89.35 (4.77)	[79.62, 99.08]	90.54 (4.77)	[80.81, 100.27]	31.09	1.43	0.25
Upper_Extremity_Scale_Normative_Score	Time	IG	43.99 (5.14)	[33.51, 54.47]	44.71 (5.14)	[34.23, 55.19]	46.06 (5.14)	[35.58, 56.54]	52.68 (5.14)	[42.20, 63.16]	31.09	1.96	0.14
	Time × Group	IAG	47.52 (4.06)	[39.24, 55.80]	49.14 (4.06)	[40.86, 57.42]	47.77 (4.15)	[39.31, 56.23]	48.81 (4.15)	[40.35, 57.27]	31.09	1.43	0.25
**PODCI**													
Transfer_Basic_Mobility_Scale_Standardized_Mean	Time	IG	85.76 (3.66)	[78.30, 93.22]	89.39 (3.66)	[81.93, 96.85]	90.00 (3.66)	[82.54, 97.46]	91.82 (3.66)	[84.36, 99.28]	31.06	3.73	0.02
	Time × Group	IAG	94.51 (2.89)	[88.62, 100.40]	92.90 (2.89)	[87.01, 98.79]	94.01 (2.94)	[88.01, 100.01]	96.61 (2.94)	[90.61, 102.61]	31.06	1.82	0.16
Transfer_Basic_Mobility_Scale_Normative_Score	Time	IG	27.93 (6.42)	[14.84, 41.02]	34.31 (6.42)	[21.22, 47.40]	35.38 (6.42)	[22.29, 48.47]	38.57 (6.42)	[25.48, 51.66]	31.06	3.73	**0.02**
	Time × Group	IAG	43.29 (5.08)	[32.93, 53.65]	40.46 (5.08)	[30.10, 50.82]	42.42 (5.15)	[31.92, 52.92]	46.97 (5.15)	[36.47, 57.47]	31.06	1.82	0.16
Sports_and_Physical_Functioning_Scale_Standardized_Mean	Time	IG	69.10 (9.21)	[50.32, 87.88]	81.39 (9.21)	[62.61, 100.17]	72.18 (9.21)	[53.40, 90.96]	76.53 (9.21)	[57.75, 95.31]	31.02	0.56	0.65
	Time × Group	IAG	77.60 (7.28)	[62.75, 92.45]	73.04 (7.28)	[58.19, 87.89]	76.25 (7.41)	[61.14, 91.36]	76.51 (7.41)	[61.40, 91.62]	31.02	2.13	0.12
Sports_and_Physical_Functioning_Scale_Normative_Score	Time	IG	27.16 (8.63)	[9.56, 44.76]	39.18 (8.63)	[21.58, 56.78]	30.17 (8.63)	[12.57, 47.77]	34.42 (8.63)	[16.82, 52.02]	31.01	0.57	0.64
	Time × Group	IAG	35.47 (6.83)	[21.54, 49.40]	31.49 (6.83)	[17.56, 45.42]	36.41 (6.95)	[22.24, 50.58]	34.38 (6.95)	[20.21, 48.55]	31.01	2.47	0.08
Pain_Comfort_Scale_Standardized_Mean	Time	IG	92.56 (9.81)	[72.55, 112.57]	92.00 (9.81)	[71.99, 112.01]	90.89 (9.81)	[70.88, 110.90]	92.56 (9.81)	[72.55, 112.57]	30.91	1.00	0.41
	Time × Group	IAG	71.32 (7.76)	[55.49, 87.15]	69.86 (7.76)	[54.03, 85.69]	79.33 (7.93)	[63.15, 95.51]	82.27 (7.93)	[66.09, 98.45]	30.91	1.11	0.36
Pain_Comfort_Scale_Normative_Score	Time	IG	50.07 (6.94)	[35.91, 64.23]	49.67 (6.94)	[35.51, 63.83]	48.86 (6.94)	[34.70, 63.02]	50.07 (6.94)	[35.91, 64.23]	30.91	1.22	0.32
	Time × Group	IAG	34.64 (5.49)	[23.44, 45.84]	33.58 (5.49)	[22.38, 44.78]	41.64 (5.60)	[30.22, 53.06]	42.62 (5.60)	[31.20, 54.04]	30.91	1.41	0.26
Happiness_Scale_Standardized_Mean	Time	IG	84.50 (7.59)	[69.02, 99.98]	88.50 (7.59)	[73.02, 103.98]	91.00 (7.59)	[75.52, 106.48]	91.00 (7.59)	[75.52, 106.48]	31.02	1.88	0.15
	Time × Group	IAG	76.87 (6.00)	[64.63, 89.11]	78.12 (6.00)	[65.88, 90.36]	83.69 (6.11)	[71.23, 96.15]	79.40 (6.11)	[66.94, 91.86]	31.02	0.26	0.85
Happiness_Scale_Normative_Score	Time	IG	46.25 (5.38)	[35.28, 57.22]	49.08 (5.38)	[38.11, 60.05]	50.85 (5.38)	[39.88, 61.82]	50.85 (5.38)	[39.88, 61.82]	31.02	1.88	0.15
	Time × Group	IAG	40.84 (4.25)	[32.17, 49.51]	41.73 (4.25)	[33.06, 50.40]	45.67 (4.33)	[36.84, 54.50]	42.64 (4.33)	[33.81, 51.47]	31.02	0.26	0.85
GLOBAL_FUNCTIONING_SCALE_Mean_of_Standardized_Mean	Time	IG	83.10 (5.84)	[71.19, 95.01]	87.15 (5.84)	[75.24, 99.06]	85.11 (5.84)	[73.20, 97.02]	88.98 (5.84)	[77.07, 100.89]	30.97	1.99	0.14
	Time × Group	IAG	83.12 (4.61)	[73.72, 92.52]	81.68 (4.61)	[72.28, 91.08]	84.83 (4.68)	[75.28, 94.38]	86.57 (4.68)	[77.02, 96.12]	30.97	0.86	0.47
GLOBAL_FUNCTIONING_SCALE_Normative_Score	Time	IG	35.43 (7.58)	[19.97, 50.89]	40.91 (7.58)	[25.45, 56.37]	38.15 (7.58)	[22.69, 53.61]	43.37 (7.58)	[27.91, 58.83]	30.96	2.39	0.09
	Time × Group	IAG	35.45 (5.99)	[23.23, 47.67]	33.51 (5.99)	[21.29, 45.73]	39.69 (6.07)	[27.31, 52.07]	40.14 (6.07)	[27.76, 52.52]	30.96	1.33	0.28

**Table 5:** Gait parameters in children with spastic cerebral palsy. Gait data was time-normalized to gait cycles. Data are presented as the mean (SD). Bold numbers: significant difference (*p* < 0.05).

Parameter	Effect	Group	FAM	95% CI	PRE	95% CI	POST	95% CI	FOLLOW	95% CI	DoF	F	Sig.
IC_Ankle [°]	Time	IG	−11.54 (6.07)	[−24.21, 1.13]	−12.60 (6.07)	[−25.27, 0.07]	−2.69 (6.07)	[−15.36, 9.98]	−1.29 (6.07)	[−13.96, 11.38]	19.69	1.38	0.28
	Time × Group	IAG	−6.37 (4.29)	[−15.33, 2.59]	−9.95 (4.29)	[−18.91, −0.99]	−6.31 (4.29)	[−15.27, 2.65]	−9.79 (4.56)	[−19.31, −0.27]	19.69	1.32	0.30
MaxDorsi_Ankle [°]	Time	IG	2.38 (3.63)	[−5.19, 9.95]	3.66 (3.63)	[−3.91, 11.23]	10.09 (3.63)	[2.52, 17.66]	10.03 (3.63)	[2.46, 17.60]	20.21	1.65	0.21
	Time × Group	IAG	9.30 (2.57)	[3.94, 14.66]	6.92 (2.57)	[1.56, 12.28]	10.41 (2.57)	[5.05, 15.77]	6.99 (2.76)	[1.24, 12.74]	20.21	1.33	0.29
MaxDorsi_Time [% GC]	Time	IG	36.33 (7.96)	[19.73, 52.93]	16.00 (7.96)	[−0.60, 32.60]	37.00 (7.96)	[20.40, 53.60]	37.67 (7.96)	[21.07, 54.27]	20.15	1.64	0.21
	Time × Group	IAG	44.33 (5.63)	[32.59, 56.07]	38.17 (5.63)	[26.43, 49.91]	34.33 (5.63)	[22.59, 46.07]	39.84 (6.15)	[27.02, 52.66]	20.15	1.36	0.28
MaxPlantar_Ankle [°]	Time	IG	−22.25 (6.67)	[−36.16, −8.34]	−20.97 (6.67)	[−34.88, −7.06]	−11.76 (6.67)	[−25.67, 2.15]	−9.57 (6.67)	[−23.48, 4.34]	20.08	1.60	0.22
	Time × Group	IAG	−14.36 (4.71)	[−24.18, −4.54]	−17.49 (4.71)	[−27.31, −7.67]	−10.68 (4.71)	[−20.50, −0.86]	−14.59 (5.06)	[−25.14, −4.04]	20.08	0.66	0.59
MaxPlantar_Time [% GC]	Time	IG	67.00 (9.51)	[47.16, 86.84]	66.00 (9.51)	[46.16, 85.84]	65.33 (9.51)	[45.49, 85.17]	77.67 (9.51)	[57.83, 97.51]	19.98	0.57	0.64
	Time × Group	IAG	81.67 (6.72)	[67.65, 95.69]	83.00 (6.72)	[68.98, 97.02]	82.00 (6.72)	[67.98, 96.02]	82.13 (7.06)	[67.40, 96.86]	19.98	0.60	0.62
MaxDorsi_swing_Ankle [°]	Time	IG	−7.90 (5.56)	[−19.50, 3.70]	−7.73 (5.56)	[−19.33, 3.87]	0.99 (5.56)	[−10.61, 12.59]	2.76 (5.56)	[−8.84, 14.36]	20.05	1.33	0.29
	Time × Group	IAG	0.40 (3.93)	[−7.80, 8.60]	−1.36 (3.93)	[−9.56, 6.84]	2.09 (3.93)	[−6.11, 10.29]	−0.77 (4.22)	[−9.57, 8.03]	20.05	0.93	0.44
MaxDorsi_swing_Time [% GC]	Time	IG	88.00 (6.38)	[74.68, 101.32]	86.67 (6.38)	[73.35, 99.99]	91.33 (6.38)	[78.01, 104.65]	86.00 (6.38)	[72.68, 99.32]	19.84	0.89	0.46
	Time × Group	IAG	78.67 (4.51)	[69.26, 88.08]	82.33 (4.51)	[72.92, 91.74]	82.33 (4.51)	[72.92, 91.74]	79.81 (4.64)	[70.13, 89.49]	19.84	0.41	0.75
ROM_Ankle [°]	Time	IG	24.63 (4.89)	[14.43, 34.83]	24.63 (4.89)	[14.43, 34.83]	21.92 (4.89)	[11.72, 32.12]	19.60 (4.89)	[9.40, 29.80]	20.08	0.81	0.51
	Time × Group	IAG	23.67 (3.46)	[16.45, 30.89]	24.41 (3.46)	[17.19, 31.63]	21.09 (3.46)	[13.87, 28.31]	21.70 (3.66)	[14.07, 29.33]	20.08	0.11	0.95
MaxPower [W/kg]	Time	IG	1.49 (0.29)	[0.85, 2.13]	1.84 (0.29)	[1.20, 2.48]	1.50 (0.29)	[0.86, 2.14]	1.78 (0.29)	[1.14, 2.42]	11.05	0.26	0.85
	Time × Group	IAG	1.58 (0.23)	[1.07, 2.09]	1.01 (0.21)	[0.55, 1.47]	1.32 (0.21)	[0.86, 1.78]	1.31 (0.21)	[0.85, 1.77]	11.05	2.06	0.16
MaxPower_Time [% GC]	Time	IG	39.00 (6.45)	[24.84, 53.16]	37.00 (6.45)	[22.84, 51.16]	53.00 (6.45)	[38.84, 67.16]	53.00 (6.45)	[38.84, 67.16]	11.24	2.18	0.15
	Time × Group	IAG	54.61 (5.11)	[43.39, 65.83]	55.25 (4.56)	[45.24, 65.26]	56.50 (4.56)	[46.49, 66.51]	56.00 (4.56)	[45.99, 66.01]	11.24	1.57	0.25
MaxPower_Stance [W/kg]	Time	IG	0.93 (0.35)	[0.15, 1.71]	1.17 (0.35)	[0.39, 1.95]	0.48 (0.35)	[−0.30, 1.26]	0.51 (0.35)	[−0.27, 1.29]	10.10	0.89	0.48
	Time × Group	IAG	0.43 (0.29)	[−0.22, 1.08]	0.23 (0.25)	[−0.33, 0.79]	0.23 (0.25)	[−0.33, 0.79]	0.14 (0.25)	[−0.42, 0.70]	10.10	0.48	0.70
MaxPower_Stance_Time [% GC]	Time	IG	23.00 (7.98)	[5.42, 40.58]	24.50 (7.98)	[6.92, 42.08]	18.50 (7.98)	[0.92, 36.08]	37.00 (7.98)	[19.42, 54.58]	10.92	2.88	0.09
	Time × Group	IAG	40.00 (6.29)	[26.14, 53.86]	29.25 (5.64)	[16.83, 41.67]	30.75 (5.64)	[18.33, 43.17]	41.25 (5.64)	[28.83, 53.67]	10.92	0.63	0.61
IC_Knee [°]	Time	IG	18.81 (4.44)	[9.58, 28.04]	14.08 (4.44)	[4.85, 23.31]	11.05 (4.44)	[1.82, 20.28]	11.21 (4.44)	[1.98, 20.44]	21.00	3.08	**0.05**
	Time × Group	IAG	12.02 (3.14)	[5.49, 18.55]	12.39 (3.14)	[5.86, 18.92]	9.13 (3.14)	[2.60, 15.66]	8.06 (3.14)	[1.53, 14.59]	21.00	0.57	0.64
MaxExtension_Knee [°]	Time	IG	−2.49 (4.62)	[−12.10, 7.12]	−4.58 (4.62)	[−14.19, 5.03]	−1.84 (4.62)	[−11.45, 7.77]	0.08 (4.62)	[−9.53, 9.69]	21.00	0.45	0.72
	Time × Group	IAG	8.06 (3.26)	[1.28, 14.84]	6.92 (3.26)	[0.14, 13.70]	8.32 (3.26)	[1.54, 15.10]	5.51 (3.26)	[−1.27, 12.29]	21.00	1.00	0.41
MaxExtension_Knee_Time [% GC]	Time	IG	40.00 (5.05)	[29.50, 50.50]	38.00 (5.05)	[27.50, 48.50]	37.67 (5.05)	[27.17, 48.17]	41.00 (5.05)	[30.50, 51.50]	21.00	0.53	0.67
	Time × Group	IAG	39.17 (3.57)	[31.75, 46.59]	38.83 (3.57)	[31.41, 46.25]	38.50 (3.57)	[31.08, 45.92]	42.33 (3.57)	[34.91, 49.75]	21.00	0.05	0.99
MaxFlexion_Knee [°]	Time	IG	54.12 (5.55)	[42.58, 65.66]	50.94 (5.55)	[39.40, 62.48]	54.05 (5.55)	[42.51, 65.59]	54.27 (5.55)	[42.73, 65.81]	21.00	1.17	0.34
	Time × Group	IAG	61.72 (3.93)	[53.55, 69.89]	61.15 (3.93)	[52.98, 69.32]	60.71 (3.93)	[52.54, 68.88]	54.91 (3.93)	[46.74, 63.08]	21.00	2.17	0.12
MaxFlexion_Knee_Time [% GC]	Time	IG	76.67 (1.19)	[74.20, 79.14]	76.00 (1.19)	[73.53, 78.47]	76.33 (1.19)	[73.86, 78.80]	76.67 (1.19)	[74.20, 79.14]	21.00	0.55	0.65
	Time × Group	IAG	73.33 (0.84)	[71.58, 75.08]	75.67 (0.84)	[73.92, 77.42]	74.17 (0.84)	[72.42, 75.92]	75.00 (0.84)	[73.25, 76.75]	21.00	1.20	0.34
IC_HindfootTibia [°]	Time	IG	−9.00 (3.87)	[−17.10, −0.90]	−8.87 (3.87)	[−16.97, −0.77]	−0.36 (3.87)	[−8.46, 7.74]	−0.53 (3.87)	[−8.63, 7.57]	18.98	3.34	**0.04**
	Time × Group	IAG	−6.13 (3.00)	[−12.41, 0.15]	−6.08 (2.74)	[−11.82, −0.34]	−4.79 (2.74)	[−10.53, 0.95]	−8.16 (2.74)	[−13.90, −2.42]	18.98	3.87	**0.03**
MaxDorsi_HindfootTibia [°]	Time	IG	1.39 (3.00)	[−4.88, 7.66]	2.35 (3.00)	[−3.92, 8.62]	9.27 (3.00)	[3.00, 15.54]	7.61 (3.00)	[1.34, 13.88]	19.34	3.12	**0.05**
	Time × Group	IAG	4.87 (2.45)	[−0.25, 9.99]	4.76 (2.12)	[0.33, 9.19]	7.70 (2.12)	[3.27, 12.13]	2.94 (2.12)	[−1.49, 7.37]	19.34	1.83	0.18
MaxDorsi_HindfootTibia_Time [% GC]	Time	IG	25.67 (8.29)	[8.33, 43.01]	16.00 (8.29)	[−1.34, 33.34]	35.67 (8.29)	[18.33, 53.01]	36.33 (8.29)	[18.99, 53.67]	19.13	2.58	0.08
	Time × Group	IAG	40.71 (6.69)	[26.71, 54.71]	30.50 (5.86)	[18.24, 42.76]	31.17 (5.86)	[18.91, 43.43]	36.00 (5.86)	[23.74, 48.26]	19.13	1.95	0.12
MaxPlantar_HindfootTibia [°]	Time	IG	−16.10 (4.79)	[−26.12, −6.08]	−15.06 (4.79)	[−25.08, −5.04]	−6.60 (4.79)	[−16.62, 3.42]	−6.97 (4.79)	[−16.99, 3.05]	19.12	2.50	0.09
	Time × Group	IAG	−12.00 (3.87)	[−20.10, −3.90]	−12.49 (3.39)	[−19.58, −5.40]	−7.75 (3.39)	[−14.84, −0.66]	−12.74 (3.39)	[−19.83, −5.65]	19.12	1.10	0.37
MaxPlantar_HindfootTibia_Time [% GC]	Time	IG	66.00 (9.60)	[45.90, 86.10]	77.67 (9.60)	[57.57, 97.77]	69.00 (9.60)	[48.90, 89.10]	77.67 (9.60)	[57.57, 97.77]	18.86	0.61	0.62
	Time × Group	IAG	82.59 (8.05)	[65.73, 99.45]	83.67 (6.79)	[69.45, 97.89]	77.67 (6.79)	[63.45, 91.89]	82.67 (6.79)	[68.45, 96.89]	18.86	0.26	0.85
MaxDorsi_swing_HindfootTibia [°]	Time	IG	−6.04 (3.64)	[−13.65, 1.57]	−4.42 (3.64)	[−12.03, 3.19]	3.32 (3.64)	[−4.29, 10.93]	3.34 (3.64)	[−4.27, 10.95]	19.17	3.59	**0.03**
	Time × Group	IAG	−2.31 (2.89)	[−8.36, 3.74]	−0.41 (2.57)	[−5.79, 4.97]	1.07 (2.57)	[−4.31, 6.45]	−2.54 (2.57)	[−7.92, 2.84]	19.17	2.68	0.08
MaxDorsi_swing_HindfootTibia_Time [% GC]	Time	IG	90.00 (6.27)	[76.87, 103.13]	86.67 (6.27)	[73.54, 99.80]	93.00 (6.27)	[79.87, 106.13]	85.67 (6.27)	[72.54, 98.80]	18.80	0.89	0.47
	Time × Group	IAG	77.05 (4.82)	[66.95, 87.15]	83.83 (4.44)	[74.53, 93.13]	83.00 (4.44)	[73.70, 92.30]	82.83 (4.44)	[73.53, 92.13]	18.80	1.47	0.25
ROM_HindfootTibia [°]	Time	IG	17.49 (3.76)	[9.62, 25.36]	17.41 (3.76)	[9.54, 25.28]	16.74 (3.76)	[8.87, 24.61]	14.58 (3.76)	[6.71, 22.45]	18.96	0.38	0.77
	Time × Group	IAG	16.79 (3.05)	[10.41, 23.17]	17.34 (2.66)	[11.77, 22.91]	15.45 (2.66)	[9.88, 21.02]	15.75 (2.66)	[10.18, 21.32]	18.96	0.10	0.96
IC_ForefootHindfoot [°]	Time	IG	−3.02 (4.43)	[−12.35, 6.31]	−4.20 (4.43)	[−13.53, 5.13]	−5.55 (4.43)	[−14.88, 3.78]	−3.89 (4.43)	[−13.22, 5.44]	17.38	0.20	0.90
	Time × Group	IAG	−1.89 (3.76)	[−9.81, 6.03]	−4.97 (3.39)	[−12.11, 2.17]	−1.64 (3.41)	[−8.82, 5.54]	−0.67 (3.41)	[−7.85, 6.51]	17.38	0.19	0.90
MaxDorsi_ForefootHindfoot [°]	Time	IG	5.46 (3.42)	[−1.76, 12.68]	5.27 (3.42)	[−1.95, 12.49]	2.49 (3.42)	[−4.73, 9.71]	4.43 (3.42)	[−2.79, 11.65]	16.74	0.26	0.85
	Time × Group	IAG	7.68 (2.92)	[1.51, 13.85]	5.06 (2.63)	[−0.50, 10.62]	9.14 (2.64)	[3.56, 14.72]	10.49 (2.64)	[4.91, 16.07]	16.74	0.71	0.56
MaxDorsi_ForefootHindfoot_Time [% GC]	Time	IG	36.33 (7.82)	[19.82, 52.84]	34.67 (7.82)	[18.16, 51.18]	38.00 (7.82)	[21.49, 54.51]	46.67 (7.82)	[30.16, 63.18]	16.90	1.17	0.35
	Time × Group	IAG	39.97 (6.71)	[25.81, 54.13]	42.55 (6.02)	[29.84, 55.26]	36.80 (6.04)	[24.05, 49.55]	48.80 (6.04)	[36.05, 61.55]	16.90	0.18	0.91
MaxPlantar_ForefootHindfoot [°]	Time	IG	−10.79 (5.25)	[−21.84, 0.26]	−10.47 (5.25)	[−21.52, 0.58]	−9.87 (5.25)	[−20.92, 1.18]	−7.71 (5.25)	[−18.76, 3.34]	17.52	0.40	0.76
	Time × Group	IAG	−5.71 (4.51)	[−15.20, 3.78]	−8.80 (4.05)	[−17.33, −0.27]	−3.89 (4.06)	[−12.44, 4.66]	−2.56 (4.06)	[−11.11, 5.99]	17.52	0.10	0.96
MaxPlantar_ForefootHindfoot_Time [% GC]	Time	IG	67.00 (8.37)	[49.36, 84.64]	77.00 (8.37)	[59.36, 94.64]	68.67 (8.37)	[51.03, 86.31]	76.67 (8.37)	[59.03, 94.31]	17.23	0.13	0.94
	Time × Group	IAG	87.70 (6.93)	[73.09, 102.31]	75.74 (6.32)	[62.42, 89.06]	79.13 (6.38)	[65.68, 92.58]	75.13 (6.38)	[61.68, 88.58]	17.23	1.69	0.21
MaxDorsi_swing_ForefootHindfoot [°]	Time	IG	−1.54 (4.05)	[−10.07, 6.99]	−4.31 (4.05)	[−12.84, 4.22]	−3.05 (4.05)	[−11.58, 5.48]	−2.73 (4.05)	[−11.26, 5.80]	17.39	0.34	0.80
	Time × Group	IAG	1.11 (3.49)	[−6.24, 8.46]	−0.62 (3.13)	[−7.21, 5.97]	3.03 (3.13)	[−3.56, 9.62]	4.39 (3.13)	[−2.20, 10.98]	17.39	0.18	0.91
MaxDorsi_swing_ForefootHindfoot_Time [% GC]	Time	IG	80.67 (7.83)	[64.08, 97.26]	81.67 (7.83)	[65.08, 98.26]	75.33 (7.83)	[58.74, 91.92]	81.33 (7.83)	[64.74, 97.92]	16.13	0.10	0.96
	Time × Group	IAG	76.51 (6.60)	[62.53, 90.49]	74.79 (5.97)	[62.14, 87.44]	81.35 (6.01)	[68.62, 94.08]	70.35 (6.01)	[57.62, 83.08]	16.13	0.80	0.51
ROM_ForefootHindfoot [°]	Time	IG	16.24 (2.87)	[10.19, 22.29]	15.74 (2.87)	[9.69, 21.79]	12.35 (2.87)	[6.30, 18.40]	12.14 (2.87)	[6.09, 18.19]	17.26	0.68	0.58
	Time × Group	IAG	13.51 (2.43)	[8.39, 18.63]	13.87 (2.20)	[9.23, 18.51]	12.98 (2.21)	[8.32, 17.64]	13.00 (2.21)	[8.34, 17.66]	17.26	0.33	0.80

## Data Availability

Data can be made available on reasonable request.

## Supplementary Materials

The following supporting information can be downloaded at: https://www.mdpi.com/article/10.3390/jcm15103869/s1, CONSORT 2025 checklist of information to include when reporting a randomised trial [30].

## Abbreviations

The following abbreviations are used in this manuscript:

AFO	Ankle-foot orthosis
CAST	Calibrated anatomical systems technique
CP	Cerebral palsy
FS	Foot shell
GMFCS	Gross motor function classification system
GOAL	Gait Outcomes Assessment List
IC	Initial contact
IG	Immobilization group
IAG	Immobilization/Activity group
IMVC	Isometric maximum voluntary contraction
MDF	Maximum dorsiflexion
MPF	Maximum plantarflexion
PODCI	Paediatric Outcome Data Collection Instrument
RoM	Range of Motion
SCALE	Selective Control Assessment of the Lower Extremity
